# Medical Students’ Perception and Perceived Value of Peer Learning in Undergraduate Clinical Skill Development and Assessment: Mixed Methods Study

**DOI:** 10.2196/25875

**Published:** 2021-07-13

**Authors:** Shaikha Alzaabi, Mohammed Nasaif, Amar Hassan Khamis, Farah Otaki, Nabil Zary, Sharon Mascarenhas

**Affiliations:** 1 Department of Clinical Sciences, College of Medicine Mohammed Bin Rashid University of Medicine and Health Sciences Dubai United Arab Emirates; 2 Training and Development Center Ministry of Health Sharjah United Arab Emirates; 3 Institute for Excellence in Health Professions Education Mohammed Bin Rashid University of Medicine and Health Sciences Dubai United Arab Emirates

**Keywords:** peer learning, assessment, empowerment, undergraduate, medical students, self-regulated learning

## Abstract

**Background:**

The effectiveness of peer learning in clinical skill development is well recognized and researched, given the many benefits gained such as enhanced learning, alleviation of the burden on faculty, and early development of teaching skills for future doctors. However, little is known in terms of its effectiveness as an assessment tool and the extent to which peer assessment can be relied upon in the absence of faculty support.

**Objective:**

This study was conducted to assess medical students’ perception toward peer learning, which is based on self-regulated learning as a tool of assessment, and to compare peer evaluation with faculty evaluation of clinical skill performance.

**Methods:**

A cohort of 36 third-year medical students were exposed to peer learning (same-level) in clinical skills education for 3 months. A convergent mixed methods approach was adapted to collect data from 3 sources, namely, students’ perception of peer learning, performance scores, and reflective observational analysis. A 5-point Likert-type scale was used to assess students’ (n=28) perception on the value of peer learning. The students were asked to assess their peers by using a preset checklist on clinical skill performance, and scores were compared to faculty assessment scores. Reflective observational data were collected from observing video recordings of some of the peer learning sessions. The findings from all 3 sources were integrated using joint display analysis.

**Results:**

Out of 28 students, 25 students completed the survey and 20 students perceived peer learning as valuable in clinical skills education. The mean score of peer assessment was higher than that of faculty assessment. There was a significant difference in student performance between supervised teaching and peer learning groups (*P*=.003). Most students focused on the mastery of skill with little attention to the technique’s quality. Further, students were unable to appreciate the relevance of the potential clinical findings of physical examination.

**Conclusions:**

Peer learning in clinical skills education, based on self-regulated learning, empowers students to develop a more responsible approach toward their education. However, peer assessment is insufficient to evaluate clinical skill performance in the absence of faculty support. Therefore, we recommend that peer learning activities be preceded by supervised faculty-taught sessions.

## Introduction

Peer learning is defined as “people from similar social groupings, who are not professional teachers, helping each other learn, and by so doing, learning themselves” [1]. Peer learning can be categorized into (1) same-level peer learning where students at equal academic levels discus and study the materials together and (2) cross-level peer learning where students’ academic levels diverge [2]. Peer learning is rapidly gaining acceptance and there are supporting evidences for peer learning in clinical skill development worldwide. An objective structured clinical examination is a complex competency assessment that assesses the cognitive knowledge as well as the psychomotor skills of clinicians. The clinician needs to recall all the steps in the correct and most efficient order and thereafter skillfully perform each step of the investigation. Finally, the clinician needs to cognitively interpret the findings of each step independently as well as all of them together to reach a better understanding of the patient’s condition. Peer learning has various benefits in clinical skill settings, including enhanced learning, cost-effectiveness [3], and alleviation of the burden on the teaching faculty [4], where some have even proposed that it might offer a solution to the global increase in the medical student numbers in the face of faculty shortage [5]. Peer learning fosters self-regulated learning. A recent study showed that students’ ability to learn with peers has a significant positive impact on their academic achievements and significantly influences their self-regulated learning strategies [6]. This study also highlights the importance of facilitating the development of students’ self-regulated learning and peer learning competencies in blended learning courses.

Self-regulated learning is defined as the degree to which students are metacognitively, motivationally, and behaviorally active participants in their own learning processes [7]. There are many validated theoretical models that conceptualize self-regulated learning, an example of which is the “dual processing self-regulatory model” formulated by Boekaerts and colleagues [8], which describes the various purposes of self-regulated learning, namely, (1) expanding one’s knowledge and skills, (2) protecting one’s commitment to the learning activity, and (3) preventing threat and harm to oneself. Another example is the “triadic social cognitive model” described by Zimmerman [9] where he introduces the interplay between the environment, behavior, and person. He also conceptualized the virtuous cyclical phases of self-regulated learning that start with forethought, followed by performance, and finally, self-reflection. By fostering self-regulated learning, peer learning provides the students with a sense of ownership [10]. This offers them an opportunity to develop the skills and professional attributes needed for teaching, assessment, and feedback. These skills and attributes are essential to nurturing a life-long culture of learning and teaching that is vital to their future roles as clinicians, especially if they decide to work in academic contexts [5,11].

Although peer learning in formative settings has been widely explored in the literature, there has been less focus on peer assessment. Despite the benefits that peer assessment offers, in terms of the development of self-regulation and self-monitoring in lifelong learning [10], it is still unclear to which extent it can be relied upon in the absence of faculty support. Accordingly, this study investigates, from the self-regulated learning perspective, the experience of undergraduate medical students concerning the application of peer learning in acquiring clinical skills. The research questions in this study are as follows:

1. How does students’ assessments of their peers compare to that of faculty?
2. How does the performance of the students receiving supervised learning compare to that of the students receiving peer learning (as assessed by the faculty in both cases)?
3. How do students perceive peer learning?
4. How does faculty perceive peer learning and what constitutes outstanding observations, from their perspective, in terms of students’ individual level attitudes and behaviors and interactions among each other?
5. What were the highlights and limitations of the intervention under investigation and how can other similar health profession educators leverage the lessons learned to effectively integrate peer learning?

## Methods

### Context of This Study

The Foundations of Clinical Medicine is a 2-credit course offered to undergraduate medical students at the Mohammed Bin Rashid University of Medicine and Health Sciences (MBRU). It runs horizontally across the first 3 years of the Bachelor of Medicine, Bachelor of Surgery degree (MBBS), complementary to the basic sciences courses. This course introduces students to history taking, physical examination, and communication skills that are necessary to conduct a successful patient investigation, where simulation is the mainstay of learning and teaching. This study focuses on a specific intervention that was implemented in the third course of the respective horizontally integrated module. The third-year MBBS students attend a class for the corresponding course every Thursday for a duration of 15 weeks covering the first semester of the respective academic year. The cohort is usually divided into 2 groups. One group would receive supervised teaching in the morning while the other would undertake a self-study session with the option to consult from a selection of relevant resources available on their learning management system. The two groups would then switch for the alternate arrangement in the afternoon, each session lasting for a 2-hour duration.

### Ethical Considerations

The MBRU institutional review board approved this study (Reference# MBRU-IRB- 2019-017). Participation in this study was voluntary with written consent in accordance with the general regulation of the College of Medicine-Human Research Ethics Committee. The survey utilized to capture the perception of the participants was anonymous.

### Intervention

The Foundations of Clinical Medicine course delivery was modified for this study where some of the self-study was substituted by peer learning sessions. The rationale for this modification was based on the feedback received from previous cohorts that self-study sessions were not of much benefit, and most of the students would rather dedicate the time toward more practice of the clinical skills. Accordingly, the core of the learning and teaching in the respective course was modified with the objective of enabling and empowering students to leverage peer learning for practicing and, in turn, improving their clinical skills. These modifications were in alignment with the 3 phases of the cyclic model of self-regulated learning proposed by Zimmerman [12], and therefore, these peer learning sessions were designed in a way to foster self-regulated learning. Table 1 compares the old and postintervention arrangements of the course delivery for a given group of students.

**Table 1:** A comparison of the preintervention and postintervention teaching arrangements for the foundations of the clinical medicine course.

Timing	Old arrangement	New arrangement
8 AM-10 AM	Supervised teaching	Peer learning
11 AM-1 PM	Break	Break
2 PM-4 PM	Self-study	Supervised teaching

All students were initially exposed to a video demonstration of the physical examination in the form of flipped learning material. The cohort was then randomly assorted into 2 groups. The first group initially underwent peer learning as the primary modality for 1 physical examination session, which was recorded using camera videos already installed in the simulation center where the intervention was conducted, after which the first group received traditional supervised teaching. As for the second group, they initially received traditional supervised teaching, and thereafter, they underwent peer learning as the secondary modality.

### Research Design

**Figure 1:**
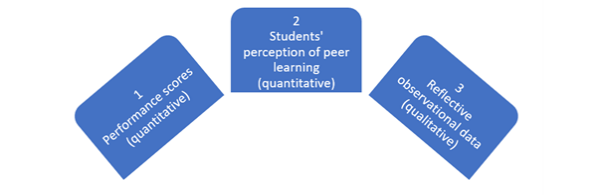
Research design. Data are triangulated from 3 sources to increase the reliability of the findings.

A convergent mixed methods approach [13-15] to research was adapted with triangulation of quantitative and qualitative data from 3 sources: performance scores, students’ perception of peer learning, and reflective observational analysis (Figure 1). The integration was conducted through joint display analysis [16].

### Data Collection and Analyses

#### Performance Scores (Quantitative)

**Figure 2:**
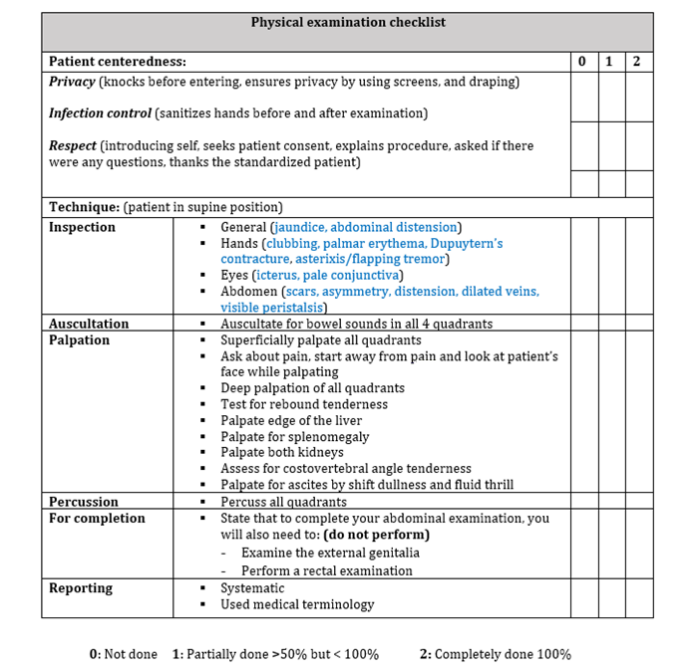
Tutor checklist for gastrointestinal examination.

**Figure 3:**
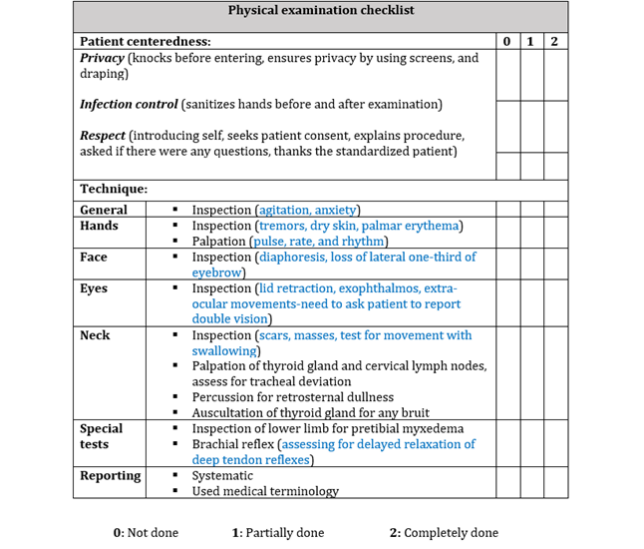
Tutor checklist for endocrine examination.

A preset checklist was used where students provided quantitative scores to assess the performance of their peers (Figures 2 and 3). The checklist is composed of 2 sections: (1) patient centeredness (to assess soft skills, including confidentiality and communication), and (2) technique performance consisting of a list of psychomotor steps to be performed by the students in the sequence outlined, including systematic reporting of findings by using appropriate medical terminology. The students were asked to assess their peers’ performance against a dichotomous variable (done or not done). Two researchers, SAZ and MN (referred to as Faculty 1 and Faculty 2, respectively), used the same checklist to evaluate the students’ performance both for supervised learning and when the students were undergoing peer learning as the primary modality (the same researchers did the latter, each independently, while observing the abovementioned recordings each at their own pace over 2 weeks). One of the researchers is an Internal Medicine physician and a faculty member at MBRU, and she coordinated and led the learning and teaching of the course under investigation. The other researcher is an Emergency Department physician who is specialized in the continuous learning and development of health care professionals. The scores were collated and analyzed using SPSS statistics version 25 (IBM Corp) to address the abovementioned research questions. Kruskal-Wallis test was used to compare faculty and peer assessment scores, and a two-tailed *t* test was performed to compare traditional supervised teaching method and peer learning method. A *P* value less than .05 was used as the level of significance in both tests.

#### Students’ Perception of Peer Learning (Quantitative)

A 5-point Likert-type scale (1: strongly disagree, 2: disagree, 3: not sure, 4: agree, and 5: strongly agree) survey composed of 8 components (Table 2) was used to anonymously assess students’ perception on the value of peer learning in clinical skills education. Quantitative analyses of the data collected using the respective questionnaires were performed using SPSS statistics version 25. Cronbach alpha was used to test the reliability of the questionnaire. Factorial analysis was used to test the validity of the questionnaire. The score of the agreement was assessed by cross bonding calculation. Interitem correlation with the percentage of agreement was calculated. Mann-Whitney *U* test was used to compare the mean scores between the 2 groups. *P* values less than .05 were considered significant.

**Table 2:** Assessment of students’ perception on the value of peer learning by their ratings on survey questions on a 5-point Likert-type scale.

Questions	Likert scale score				
	1	2	3	4	5
Question 1: The objectives were covered in the peer learning sessions	□ Strongly disagree	□ Disagree	□ Not sure	□ Agree	□ Strongly agree
Question 2: Peer learning has improved my clinical skills	□ Strongly disagree	□ Disagree	□ Not sure	□ Agree	□ Strongly agree
Question 3: Peer learning sessions created a safe learning environment	□ Strongly disagree	□ Disagree	□ Not sure	□ Agree	□ Strongly agree
Question 4: I feel peer learning is useful for my Objective Structured Clinical Examination preparation	□ Strongly disagree	□ Disagree	□ Not sure	□ Agree	□ Strongly agree
Question 5: Time allotted for the peer learning sessions was adequate	□ Strongly disagree	□ Disagree	□ Not sure	□ Agree	□ Strongly agree
Question 6: Content and quality of the handout was good	□ Strongly disagree	□ Disagree	□ Not sure	□ Agree	□ Strongly agree
Question 7: I recommend the continuation of the same method in the following years	□ Strongly disagree	□ Disagree	□ Not sure	□ Agree	□ Strongly agree
Question 8: I recommend using the same method for other courses	□ Strongly disagree	□ Disagree	□ Not sure	□ Agree	□ Strongly agree

### Reflective Observational Data (Quantitative and Qualitative)

This component of the study relied on an ethnographic approach to research by using direct and unobtrusive observations. Along with quantitatively rating each student against the checklist (referred to in the quantitative performance scores), the abovementioned researchers also evaluated the students’ performances and noted down all outstanding observations (ie, qualitative data), including the attitudes and behaviors of the students on an individual level and their interactions with each other. After the completion of the data collection, quantitative data were analyzed descriptively using SPSS statistics version 25. As for the qualitative, data, researchers adapted the 6-step framework for thematic analysis initially introduced by Braun and Clarke [17]. It is recommended to use this technique in research on health professionals’ education [18], and it is frequently put into practice in this realm [19-21]. Accordingly, the researchers (independently) familiarized themselves with the data and then generated the initial codes. Thereafter, the researchers convened 2 consecutive 1-hour meetings to present the noted observations to each other, reflect upon them, and develop a common ground (in relation to the surfacing codes), which enabled effective collaboration around the searching for themes and their review. The researchers then defined and named the themes and reported upon them.

### Joint Display Analysis

Findings from all 3 concurrent analyses were merged using joint display analysis [16]. The findings from those analyses were compared (and contrasted). The areas where those findings confirmed or built upon each other were identified. The integration also created the space for contradictory findings in any one area to be highlighted and considered in conjunction with each other when undergoing meta-inferences to weave a consistent narrative out of this study’s findings [22,23].

## Results

### Performance Scores (Quantitative)

#### Comparison of Peer and Faculty Assessment

A comparison of peer assessments with faculty assessments of clinical examination skills showed that the mean score of peer evaluation was significantly higher at 18.05 (SD 2.15) out of 20 compared to 12.67 (SD 2.63) for Faculty 1 and 11.89 (SD 4.80) for Faculty 2. Both faculty members had comparable means.

#### Comparison of Supervised Teaching and Peer Learning

There was a significant difference between the assessment scores of students who received traditional supervised teaching compared to those who received peer learning as the primary teaching modality. Scores were significantly higher in the supervised groups compared to the peer learning group with a mean of 17.33 (SD 2.57) for the former and 14.20 (SD 3.25) for the latter (*P*=.003).

#### Students’ Perception of Peer Learning (Quantitative)

Of the total student population, 89% (32/36) completed and returned the questionnaire. The participants were third-year medical students aged between 19 and 29 years, and there were more female participants (24/36, 67%) than male participants (8/36, 22%). Around 47% (17/36) of the cohort’s grade point average lay between 3 and 4. The questionnaire was reliable at a Cronbach alpha score of .895. The mean score of the agreement calculated by cross bonding calculation was 31.56 (SD 6.58), corresponding to a total score of 79%, which shows “agreement.” The majority of the items’ score means were high, ranging from 3.91 to 4.22 toward “Agree,” except for question 5: “Time allotted for peer learning sessions was adequate” (Table 2) demonstrating the lowest mean at 3.03 (SD 1.492), voluntarily elaborated upon by some students with comments such as “…2 hours is too long a time for peer learning sessions…” In addition, question 8 “I recommend using the same method for other courses” had the second lowest mean at 3.75 (SD 1.136). The average interitem correlation for questions 1, 2, 3, 4, 6, and 7 (Table 2) were between 0.338 and 0.853. However, question 5 “Time allotted for the peer learning session was adequate” displayed a consistently low correlation with most questions having an interitem correlation at or below 0.300. Question 8 “I recommend using the same method for other courses” correlated poorly only with question 4 “I feel peer learning is useful for my Objective Structured Clinical Examination preparation” while correlating well with the rest of the questions. Factorial analysis of the questionnaire confirmed construct validity across all items.

### Reflective Observational Data (Qualitative)

**Figure 4:**
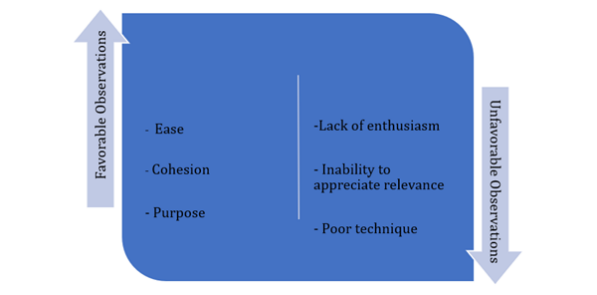
Conceptual framework of reflective observation.

The qualitative analysis conducted by the 2 abovementioned researchers resulted in a conceptual framework (Figure 4). This conceptual framework consists of 2 themes: favorable and unfavorable observations.

#### Theme 1: Favorable Observations

This theme includes the researchers’ observations that explain students’ attitudes and behaviors and ways of relating to one another that were desirable for attaining the intervention’s objective (Table 3). This theme consists of 3 categories: noticeable comfort or ease, high level of cohesion and teamwork among the students while undergoing the intervention, and urge to master skills, where students appeared to be purposefully revisiting the checklist (in a repetitive manner).

**Table 3:** Explanation for theme 1.

Observation	Interpretation
Ease in pinpointing personal and team shortcomings	Learning in a safe and relaxed environment
Evident cohesion and seeking support	Willingness and capacity to work in a team
Purposeful revisiting of the checklist	Proactiveness and urge to master skills

#### Theme 2: Unfavorable Observations

This theme includes observations that were counterproductive to attaining the objective of the intervention (Table 4). This theme encapsulated 3 categories: an overall lack of enthusiasm, inability to appreciate the relevance of potential physical findings, and poor technique while performing the set of skills.

**Table 4:** Explanation for theme 2.

Observation	Interpretation
Lack of enthusiasm	Students do not appreciate the value of these peer learning sessions
Inability to appreciate relevance	Cases where students struggle to interpret potential findings of physical examination
Poor technique	Incidences where students appeared to perform the examination, but the quality of the core technique is suboptimal

### Integration Results

**Figure 5:**
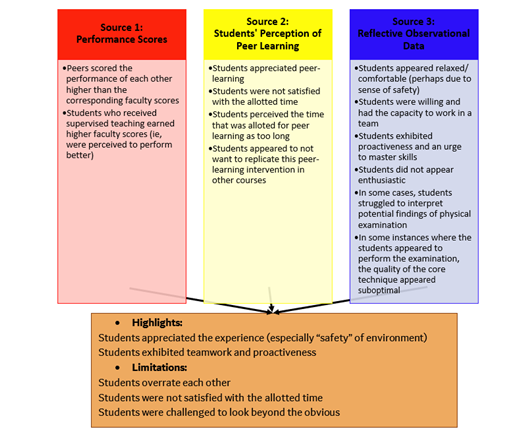
Joint display analysis based on mixing and matching of the key findings derived from the output of the concurrent analyses (each represented with a differing primary color: red, yellow, and blue). The metainferences derived are placed in a brown box to represent the mixing of the three primary colors.

The output of the 3 concurrent analyses generated findings that were all holistically considered in the iterative process of joint display analysis. Most of the findings complemented each other (as illustrated in Figure 5). To start with, in terms of highlights of the experience, the students expressed appreciation of this particular peer learning experience. Along these lines, the instructors observed that the students appeared comfortable and seemed to appreciate the safety and comfort of the encapsulating environment. The instructors also perceived that students exhibited teamwork and proactiveness. As for the limitations of the experience, the students seemed to overrate each other. Further, the students expressed dissatisfaction with the allotted time; they perceived it as too long for the purpose of the exercise. Despite their self-reported positive perception toward the exercise, the students highlighted that they would not like to replicate it in other courses. Moreover, the instructors developed the impression that the students were not enthusiastic during the experience, and (in some instances) there were aspects that seemed to challenge the students. These aspects include the core technique and the interpretation of findings.

## Discussion

### Overview of This Study

In this study, we described the implementation of peer learning as an assessment tool in clinical skills education for undergraduate medical students. The findings of this study showed how the peer learning experience under investigation is characterized by certain highlights and limitations in relation to self-regulated learning. The concept of social and cognitive congruence underlies the dynamics of peer learning and is explained by the similarity of thinking, reasoning, and social roles, which account for the successful outcomes of peer learning [5]. While participating students had an overall positive perception of peer learning, they were less objective (relative to their instructors) when evaluating their colleagues’ performance. In addition, students’ clinical skills and quality of performing the clinical examination were better under faculty supervision. However, there was clear evidence that peer learning in clinical skills fostered self-regulated learning through creation of a safe learning environment in line with the “scaffolding strategy” described by Zimmerman [9]. Accordingly, the findings of this study recommend other similar programs to integrate peer learning into undergraduate clinic skills education. Such an intervention should be designed in a way to leverage this technique’s highlights while circumventing its limitations.

### Principal Results

#### Performance Scores

Comparing peer learning with faculty assessment of clinical skill performance is essential for establishing the concurrent validity of peer assessment. Our results showed that the mean score for peer assessment was higher than that of faculty assessment. This lack of alignment between peer and faculty evaluation could be due to the assessment of a different dimension even when using the same checklist where students tend to assess recall of steps while the faculty consider the techniques in the execution of every step of the skill set to be of equal importance. The peer assessors considered the face value of the checklist, where it solely outlined the steps. As for the instructors, their expertise automatically sets them at an advantage where they “look beyond the obvious.” From this perspective, the simplistic structure (ie, design) and content of the checklist, where there is no emphasis on the expected quality of the technique, might partially account for the occurrence of this discrepancy. Another possible explanation for this misalignment could be due to the potential bias associated with students taking on the assessor’s role [24]. Moreover, our results demonstrated that students subjected to supervised teaching as the primary modality attained higher scores than the peer learning group. This was evident from the scores recorded by the faculty through direct observation of the former group and observation of video recordings for the latter group. This was further supported and can be explained by 2 unfavorable observations noted from the qualitative analysis of peer learning, namely, suboptimal quality of technique and inability to interpret the potential findings of the examination. These findings highlight the need for the faculty to support and guide students on appropriate techniques when conducting physical examinations as this is the key to eliciting physical signs in real life patient encounters. In addition, even though feedback from peers is anticipated to be much more efficient if a checklist was used [6], we believe that students require proper training before they develop the necessary competency in assessing and guiding one another. This is consistent with findings from previous studies, which show that students cannot be reliable assessors unless they receive sufficient “training and familiarity with rating criteria, resulting in higher rater agreement and internal consistency” [25]. Accordingly, it is recommended for such interventions to be designed in a way where learners go through the supervised teaching offered by experts in the subject matter and thereafter engage in peer learning. As such, supervised teaching will precede peer learning, and the benefits of peer learning, in terms of practicing and refining clinical skills, can be leveraged after covering the technical bases.

#### Perception of the Students With Regard to Peer Learning

Findings from the questionnaire demonstrated that most students had a positive perception toward peer learning, which is in line with most research findings [2,26]. However, it seems that the benefits of peer learning were limited as most students were reluctant to recommend implementing peer learning in other courses. This may be due to the students perceiving 2 hours as too long of a duration for the exercise. Another possible explanation is that all other courses are knowledge-based, and therefore, such a peer activity may not be relevant or of much benefit.

#### Reflective Observations

A general lack of enthusiasm was observed among students during the peer learning sessions. A possible explanation could be that there is not much at stake as this team activity was considered part of their formative rather than summative assessment. This attitude toward formative assessments is most probably accounted for by the lack of maturity in terms of self-regulated learning skills among our preclinical students, which is one of the many disadvantages of an exam-oriented culture [26]. Moreover, this explanation may justify the students’ underappreciation of the time dedicated to the peer learning sessions, as highlighted from their comments in the questionnaire. Pintrich [27] highlighted the importance of motivation in self-regulated learning. He perceives the steps of self-regulated learning to be (1) forethought, planning, and activation, (2) monitoring, (3) control, and (4) reaction and reflection. Each of those steps, in his opinion, has 4 different areas for regulation (cognition, motivation, behavior, and context). From this perspective, a possible solution to the observed lack of enthusiasm among participating students in this study could be to dedicate some time at the beginning of the course to better orient students to the short-term and long-term benefits of peer learning as a way of motivating them (ie, increase the perceived benefits of engaging in the exercise).

A selective learning approach dominated students’ learning behavior during peer learning sessions. The majority focused on attaining a sequential mastery of the examination steps, regardless of the quality of performance. This behavior is consistent with the predictions of the cognitive load theory [28]. The differential use of the checklist, however, between students and faculty was evident as students would tend to use it as a learning tool while the faculty would consider it more of an assessment tool. This reflects the cultural norms here in the United Arab Emirates, where there is a high level of cooperation and uncertainty avoidance. This trait was further demonstrated by competent students supporting others through repetition of skills toward mastery, which is in line with the social learning theory that describes the cooperative nature of students’ learning from each other through “modelling, instructing, and feedback” [29]. Moreover, Hadwin et al [30] discuss self-regulated learning in the context of collaborative learning, where they differentiate between coregulation and socially shared regulated learning. In the former, the regulatory actions are guided by a particular group member. As for the latter, regulatory actions emerge through a series of transactive exchanges among group members, which were clearly observed during our peer learning sessions. Another interesting observation was that the importance of relevance of any potential clinical finding did not seem to be a priority of the learning experience to most students. Consequently, despite the efforts some students invested into interpreting potential findings of the physical examination, they still, on many occasions, ended up providing misguided peer correction.

Given these findings, we feel that a subject matter expert in clinical skills education is essential for assisting students in executing the correct clinical techniques, for enabling them to appreciate the potential findings of a physical examination, and for attending to their questions and uncertainties. On a positive note, it seems that peer learning creates a safe environment for the students, which is in line with evidence from studies where students reported comfort in interacting without the pressure of competition [31] and simply feel more at ease with a peer [5]. This kind of safety in learning falls under the scaffolding strategy described by Zimmerman [9], which he considers to be a key factor in the performance phase of self-regulation, and he elaborates on the fact that “it may also help to enrich the learning experience by allowing students to dig deeper into the content and further explore.”

### Strengths and Limitations of This Study

Our study’s strength lies in 3 main features: (1) performance of the study in a live educational setting, (2) integration of data through the use of a convergent mixed methods approach to research, and (3) randomization in the cross-over part of the study.

One of this study’s main limitations is that the generalizability of the findings is limited due to the small sample size of the participants. Another limitation is that the peer assessment scores obtained were not a pure reflection of performance as students mostly used the checklist as a learning tool rather than as an assessment tool. This is, of course, in addition to the fact that peer assessment may lack objectivity due to the abovementioned potential bias, which questions its reliability in terms of assessment. Moreover, although decided for simplicity purposes, the score divisions of 1 for “done” and 0 for “not done” on the evaluation checklist did not reflect the quality of performance that is usually assessed in a broader spectrum. Finally, this intervention’s outcomes were limited to step 1 “reaction” and to a lesser degree, step 2 “learning” of Kirkpatrick’s model of evaluation [32].

### Comparison With Prior Work

Our findings of students’ positive perception toward peer learning are in line with findings of most research studies in this area [2,26]. However, it seems that our decision for students to undertake peer learning as a primary modality with no previous training might have been miscalculated as most studies ensured that peer-assisted tutors were subjected to some amount of training [33]. This might, in part, account for the misguided peer correction mentioned earlier and perhaps even the misalignment between peer and faculty evaluation of clinical skill performance. In addition, most peer learning studies focused on cross-level peer learning. In contrast, in our study, we investigated same-level peer learning to make use of advantages such as informality and practicality in terms of timetabling compared to cross-level peer learning [33]. With regards to the comparison of outcomes of clinical skill teaching by peers compared to faculty as a primary modality, the evidence in the literature is controversial as some studies reported no significant difference [2] while others concluded that students in the faculty-led teaching group required lesser time to reach the desirable outcomes [5].

### Further Work

The long-term effects of peer learning in medical education are poorly understood [5]; therefore, more robust outcome measure tools need to be developed that would go beyond the first and second levels of Kirkpatrick’s model of evaluation [18]. Moreover, we recommend future studies to tackle a larger sample size of participants for a more reliable statistical analysis and more representative findings.

### Conclusions

Our study’s findings provided evidence of acceptability and benefits of peer learning in the clinical skills education of undergraduate medical students that includes but is not limited to promoting interactive social learning. The intervention under investigation also constituted a safe learning environment for students to exercise self-regulated learning. However, peer learning is insufficient as a standalone strategy. Therefore, it needs to be preceded by supervised teaching provided by a subject matter expert for the maximum benefit to be gained. In summary, we recommend incorporating peer leaning as a secondary modality into the design of medical curricula to empower students to exercise self-regulated learning and enable them to acquire teaching and assessment skills early on in their learning trajectory that will foster a lifelong culture of teaching [26].

## Abbreviations

MBRU: Mohammed Bin Rashid University of Medicine and Health Sciences
